# A waist-to-height ratio of 0.50 marks declining physical performance in active adults

**DOI:** 10.1038/s41430-026-01798-z

**Published:** 2026-07-30

**Authors:** Adam W. Potter, Christy L. Christopoulos, Lynn Cialdella-Kam, Karl E. Friedl

**Affiliations:** 1 United States Army Research Institute of Environmental Medicine, Natick, MA USA; 2 Human Performance Branch, Training and Standards Division, Training and Education Command, United States Marine Corps, Quantico, VA USA; 3 Warfighter Performance Department, Naval Health Research Center, San Diego, CA USA

**Keywords:** Biological techniques, Anatomy

## Abstract

**Background:**

For physically demanding job standards, simple anthropometric screening tools are useful. Using military personnel as a model, this study evaluated waist-to-height ratio (WHtR) for its relationship with health and performance indicators.

**Methods:**

Data were analyzed from 2153 active-duty U.S. Marines (1421 men; 732 women). Relationships were assessed between WHtR, a circumference-based “tape test” (TT), body mass index (BMI), and a criterion measure of dual-energy X-ray absorptiometry (DXA)-derived percent body fat (%BF). Associations between WHtR and physical readiness were evaluated using composite scores from the Physical and Combat Fitness Tests (PFT, CFT), and individual performance metrics (pull-ups, crunches, 880-yard sprint time, 3-mile run time, ammo can overhead lifts).

**Results:**

WHtR was strongly correlated with DXA %BF (*r* = 0.754, *p* < 0.001, men; *r* = 0.677, *p* < 0.001, women) and moderately negatively with PFT (*r* = −0.33, *p* < 0.001) and CFT (*r* = −0.30, *p* < 0.001) scores for men. A graded decline in physical fitness scores was observed with increasing WHtR, with a clear inflection in performance outcomes occurring between WHtR values of approximately 0.48–0.50. Receiver Operating Characteristic (ROC) analysis to identify high %BF showed the TT (AUC: 0.889 men, 0.876 women) performed better than WHtR (AUC: 0.851 men, 0.848 women) and BMI.

**Conclusions:**

WHtR is a simple and effective screening tool to identify personnel at potential risk for poor performance outcomes. These findings support use of WHtR as a screening metric and a potential replacement for traditional height-weight screening tables, with a pragmatic threshold of 0.50 to identify individuals who may benefit from secondary assessment, such as from multi-frequency bioelectrical impedance analysis.

## Introduction

Physical readiness is a critical component of individual effectiveness in military, first responders, law enforcement, and other physically demanding professions. Physical performance can be periodically tested to ensure that it is being maintained; body fat testing complements the physical fitness testing by motivating exercise and nutrition habits and preventing obesity [1, 2].

The US Department of Defense (DoD) and the Marine Corps have traditionally relied on a two-tiered system of BMI screening tables, followed by circumference-based body fat estimation for those who exceed the screening weights (BMI > 25, women; BMI > 27.5, men). The actual standard is relative body fat, not weight, and this is calculated from height and a minimal set of body circumference measurements (the “tape test”, TT). These equations use abdominal circumference as a primary predictor of adiposity in men and women. This measure also serves as a proxy for central adiposity, acknowledging the association between abdominal fat, physical fitness, health risks, and military appearance [3–8]. The importance of waist circumference to health outcomes was recognized long before height-weight tables, with major insurance companies using waist circumference as the key predictor of all-cause mortality as early as 1937 [9]. While the TT approach is practical and has demonstrated acceptable performance across diverse body types including highly muscular individuals [1], it is inherently operator-dependent and requires sex-specific equations, including an additional hip circumference to correctly account for lower body fat patterning in women [2–5]. After more than 50 years of research, there is little evidence that equations to predict body from anthropometry (i.e., either skinfold thickness or body circumferences) can be meaningfully improved. These standards have been a cornerstone of military health policy for the past four decades, but limitations have motivated interest in alternate strategies [1, 2, 8, 10–13].

Waist-to-height ratio (WHtR) has emerged as a simpler anthropometric index for assessing adiposity. It requires only waist and height measurements, eliminating the need for sex-specific equations. While the military has searched for better methods of body fat assessment, WHtR could, at a minimum, replace body weight with waist circumference in the initial screening phases of the two-tiered system used by the military [14]. Height-weight (based on BMI) assessment has been a longstanding concern because body weight does not reliably predict body composition or health and performance outcomes, and can lead to inappropriate weight loss behaviors before scheduled assessments [14]. Beyond operational considerations, WHtR is widely recognized as a superior screening tool for central adiposity and cardiometabolic risk compared to BMI [6–8]. A large body of evidence supports a simple public health message: ‘keep your waist circumference to less than half your height’ [9, 15]. Large-scale meta-analyses have established that WHtR is a better predictor than BMI or waist circumference alone for diabetes, hypertension, dyslipidemia, and cardiovascular diseases [10, 15]. A WHtR of 0.50 is consistently identified as a universal, early-risk threshold, indicating when central fat accumulation may increase the likelihood of these adverse health outcomes [9–12].

This work evaluated WHtR as a replacement for the height-for-weight (i.e., BMI) screening step and its comparability to the TT for body fat assessment in the US military. Specific objectives were to: [1] evaluate the relationship between WHtR, TT, and BMI in predicting dual-energy X-ray absorptiometry (DXA)-derived %BF across sex and age groups in active-duty Marines, and [2] assess the relationship between WHtR and readiness-related outcomes, using Physical Fitness Test (PFT) and Combat Fitness Test (CFT) scores as indicators of functional performance.

## Materials and methods

### Participants

The sample represents a broad cross-section of active-duty personnel enrolled in a larger Marine Corps Body Composition Study [13]. For men (*n* = 1421), the mean age was 30.2 years (SD = 7.73, range: 18–57). For women (*n* = 732), the mean age was 29.5 years (SD = 7.27, range: 18–56). While the distribution naturally skews toward the younger demographics typical of military service, the mean age of ~30 and the wide range indicate that our findings regarding WHtR and performance are generalizable across early and mid-career life stages.

Recruitment and data collection occurred at Camp Pendleton, California; Camp Lejeune, North Carolina; and the National Capital Region (Quantico, Virginia). Prior to study-related activities, all participants provided written informed consent, and women were provided a rapid pregnancy test to establish absence of detectable pregnancy. Study approval was granted by the US Army Medical Research and Development Command (Fort Detrick, Maryland) and US Marine Corps (Quantico, Virginia) Institutional Review Boards, protocol M10873, approved March 2021.

### Study design

Each participant was interviewed and then assessed for body composition during a single-day visit (<2 h). Participants all wore athletic clothing (t-shirt and shorts) and were asked to remove all jewelry and/or foreign objects. Standing height measurements were recorded to the nearest 0.1 cm, using a calibrated stadiometer (Seca, Chino, California). Body weight measurements were recorded using a calibrated digital scale (Model 400, Angel USA, Vancouver, British Columbia, Canada). Anthropometric data were collected for circumference measures (waist, neck, hip) in triplicate to the nearest 5 mm using a standard tape measure (MyoTape, AccuFitness LLC, Denver, Colorado) in accordance with MCO 6100.10. Waist circumference (cm) was measured from three different waist/abdomen locations: Abdomen-1 (AB1) from the narrowest circumference between the bottom of the rib cage and the iliac crest, AB2 horizontal with the navel, and AB3 at the medial aspect of the iliac crest. The Marine Corps standard uses AB2 for the prediction of relative body fat in men and AB1 for women; WHO reference values are similar to AB1 but defined more precisely as midway between the lowest rib and the top of the iliac crest (i.e., not the “narrowest point”); the National Health and Nutrition Examination Survey (NHANES) uses AB3 [14, 16]. Neck circumference (cm) was measured just below the laryngeal prominence, with the tape positioned perpendicular to the long axis of the neck. Hip circumference (cm) was measured at the point of maximal protrusion of the buttocks; this was the only circumference measured over clothing, thin nylon physical fitness uniform shorts. The US Marine Corps calculates percent body fat from height and circumferences (via TT) using the DoD standard sex-specific equations developed by Hodgdon [2, 3, 17]:

Males = 86.010 × log10 [AB2 − neck (in)] − 70.041 × log10 [height (in)] + 36.76. [Converted to cm: Males = 86.010 * log10[AB2 (cm) − neck (cm)] − 70.041 * log10[height (cm)] + 30.396]

Females = 163.205 × log10 [AB1 − neck (in)] – 97.684 × log10 [height (in)] − 78.387. [Converted to cm: Females = 163.205 * log10[AB1 (cm) − neck (cm)] − 97.684 * log10[height (cm)] − 104.902]

Whole body composition measures were assessed using dual-energy X-ray absorptiometry (DXA) and algorithms (iDXA, enCORE software (version 13.5), GE Healthcare, Madison, Wisconsin). Total body relative body fat (%BF) from the DXA was used as the criterion for this analysis.

Performance data was collected based on official scores from the Physical Fitness Test (PFT) and Combat Fitness Test (CFT). Both PFT and CFT are scored on a 0–300 point scale, with performance classified as 1st Class (235–300), 2nd Class (200–234), and 3rd Class (150–199). Both PFT and CFT scores are sex- and age-adjusted (Marine Corps Order 6100.13A with Change 4, March 2022). Additionally, individual physical performance events within the PFT and CFT were evaluated: pull-ups (maximum continuous repetitions), 880-yard sprint time (seconds), 3-mile run time (seconds), and timed assessment for crunches (repetitions) and 13.61 kg (30 lbs) ammo can overhead lifts (repetitions). Existing standards for age- and sex-specific thresholds for BMI and %BF were used for comparison of metrics (Table 1). The PFT, CFT, and body composition study were conducted at separate times, including PFT and CFT typically performed at different times of the year.

**Table 1:** US Marine Corps Relative Body Fat (%BF) and body mass index (BMI) standards by sex and age group.

Sex	Age group (years)	%BF limit	BMI limit
Men	17–26	18%	27.5 (kg/m^2^)
	27–39	19%	
	40–45	20%	
	46+	21%	
Women	17–26	27%	26 (kg/m^2^)
	27–39	28%	
	40–45	29%	
	46+	30%	

### Statistical analyses

Analyses were conducted using R [Version 4.4.1; R Foundation for Statistical Computing; Vienna, Austria [18]] with packages [tidyverse, pROC, purrr, ggplot2 [19–22]]. All data is reported as mean ± standard deviation (SD) unless specified otherwise. Pearson correlation coefficients (*r*) were used to describe linear associations between anthropometric indices and DXA %BF and quantify associations between WHtR and individual performance events. Linear regression and Locally Estimated Scatterplot Smoothing (LOESS) were used to model relationships between variables. Receiver Operating Characteristic (ROC) analysis and the area under the curve (AUC) were used to evaluate the ability of WHtR, TapeT, and BMI to discriminate individuals above established %BF thresholds (Table 1).

Distribution-based comparisons were used to examine the relationships among BMI, DXA %BF, and physical performance scores (PFT and CFT) across grouped ranges of WHtR. Receiver Operating Characteristic (ROC) analyses were conducted to evaluate the ability of WHtR, TapeT, and BMI to discriminate individuals exceeding established age- and sex-specific %BF thresholds (Table 1). Area under the curve (AUC) values were calculated with 95% confidence intervals (CI) using DeLong’s method [23].

Locally Estimated Scatterplot Smoothing (LOESS) was applied to visualize potential non-linear trends and identify performance inflection points across the WHtR spectrum. Given the exploratory nature of subgroup and performance stratification analyses, results were interpreted with emphasis on effect size, consistency, and physiological plausibility rather than strict multiplicity correction.

### Ethics approval and consent to participate

All methods were performed in accordance with the relevant guidelines and regulations. Ethical approval for this study was granted by the US Army Medical Research and Development Command (Fort Detrick, Maryland) and US Marine Corps (Quantico, Virginia) Institutional Review Boards, protocol M10873, approved March 2021. Informed consent was obtained from all participants involved in this study.

## Results

### Body composition calculations

**Fig. 1:**
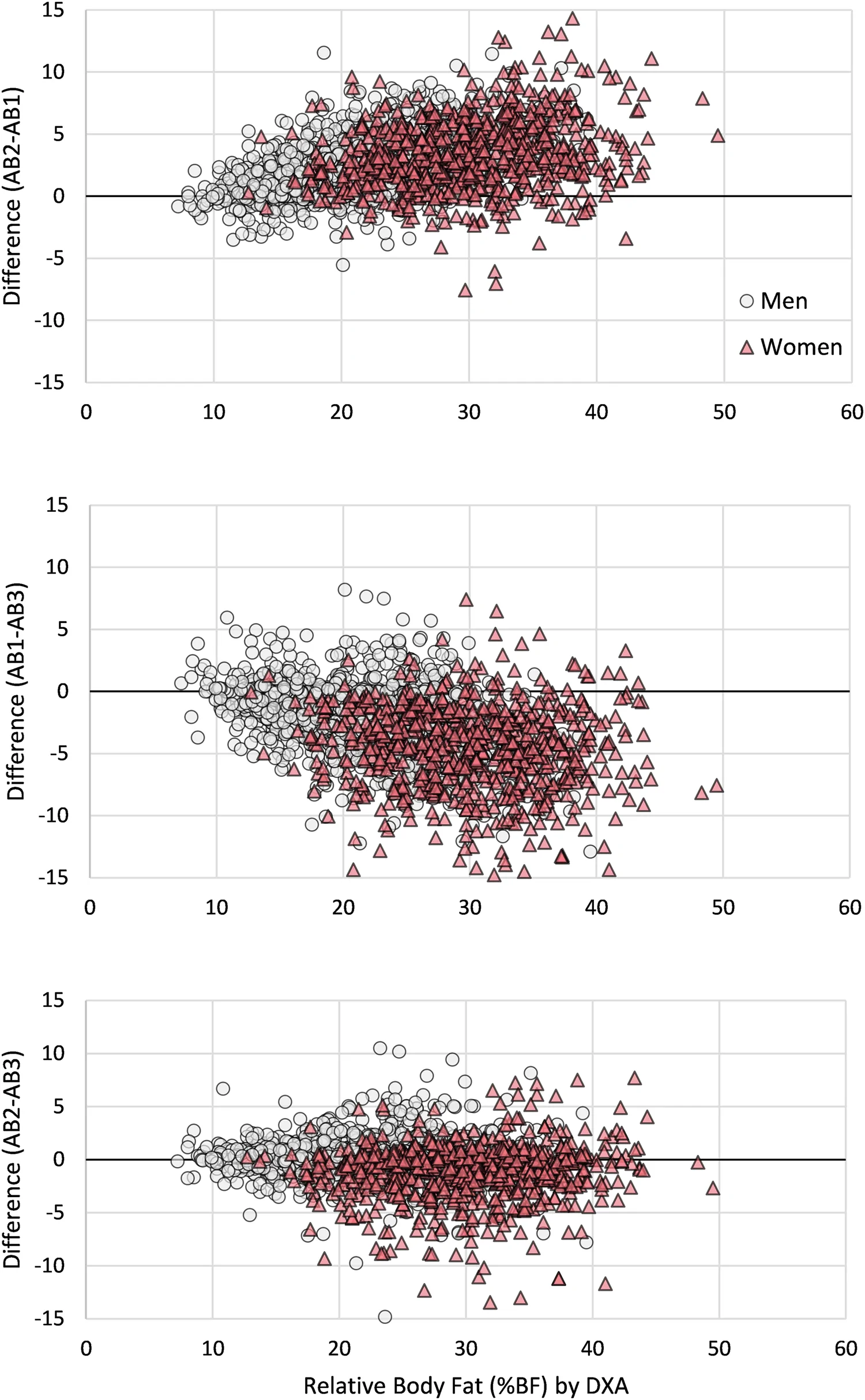
Comparison of Waist Circumference Measurement Sites. This figure shows the difference between three waist/abdomen circumference measurement sites (AB1, AB2, and AB3), plotted against the criterion measure of relative body fat percentage (%BF) from dual-energy X-ray absorptiometry (DXA). Each panel displays the difference (cm) between two measurement sites. Top Panel: Difference between AB2 (navel) and AB1 (narrowest point). Middle Panel: Difference between AB1 (narrowest point) and AB3 (iliac crest). Bottom Panel: Difference between AB2 (navel) and AB3 (iliac crest). Data points are separated by sex, with circles representing men and triangles representing women. These show the variability between different waist measurement sites.

Waist-to-Height Ratio (WHtR) was calculated using measures of waist circumference (cm)/height (cm). As the location for the measurement of waist circumference varies between large studies [16], additional analyses were conducted comparing the three different sites (AB1, AB2, and AB3, each described in the methods above). While the measures are similar, particularly within a non-obese healthy population, there are differences worth noting. Figure 1 shows the differences between the three measured sites; visually, the most similar in this sample was between AB2 and AB3, while greater differences were observed between AB1 and AB3. Based on these comparisons, AB2 was selected as the most representative waist site for WHtR calculation. Therefore, our measure of WHtR was calculated as AB2 (cm)/height (cm).

### Descriptive statistics

Summary statistics are presented in Table 2. Mean WHtR was 0.50 ± 0.05 for men and 0.46 ± 0.04 for women. Women (expectedly) displayed higher mean DXA %BF (30.1%) than men (21.9%), while mean PFT and CFT scores were comparable between sexes.

**Table 2:** Summary statistics for key variables by sex.

Variable	Men	Women
**Sample size**	**1421**	**732**
Age (years)	30.2 ± 7.7	29.5 ± 7.3
Height (cm)	176.8 ± 7.0	163.6 ± 6.8
Weight (kg)	86.0 ± 11.5	68.3 ± 9.2
BMI (kg/m^2^)	27.5 ± 3.1	25.5 ± 2.8
WHtR	0.502 ± 0.046	0.463 ± 0.040
DXA %BF	21.9 ± 6.3	30.1 ± 6.5
TapeT %BF	18.5 ± 5.2	30.5 ± 5.6
PFT	263.2 ± 49.2	255.3 ± 60.8
CFT	279.1 ± 59.1	278.7 ± 68.2
3-mile run (seconds)	1330.8 ± 317.0	1499.0 ± 416.2
880-yard sprint (seconds)	171.1 ± 44.5	207.3 ± 60.5
Pull-ups (repetitions)	19.9 ± 6.2	9.2 ± 5.2
Crunches (repetitions)	112.1 ± 44.2	105.0 ± 42.0
Ammo can overhead lifts (repetitions)	116.1 ± 27.2	76.8 ± 23.6

### Correlation and regression analysis

**Fig. 2:**
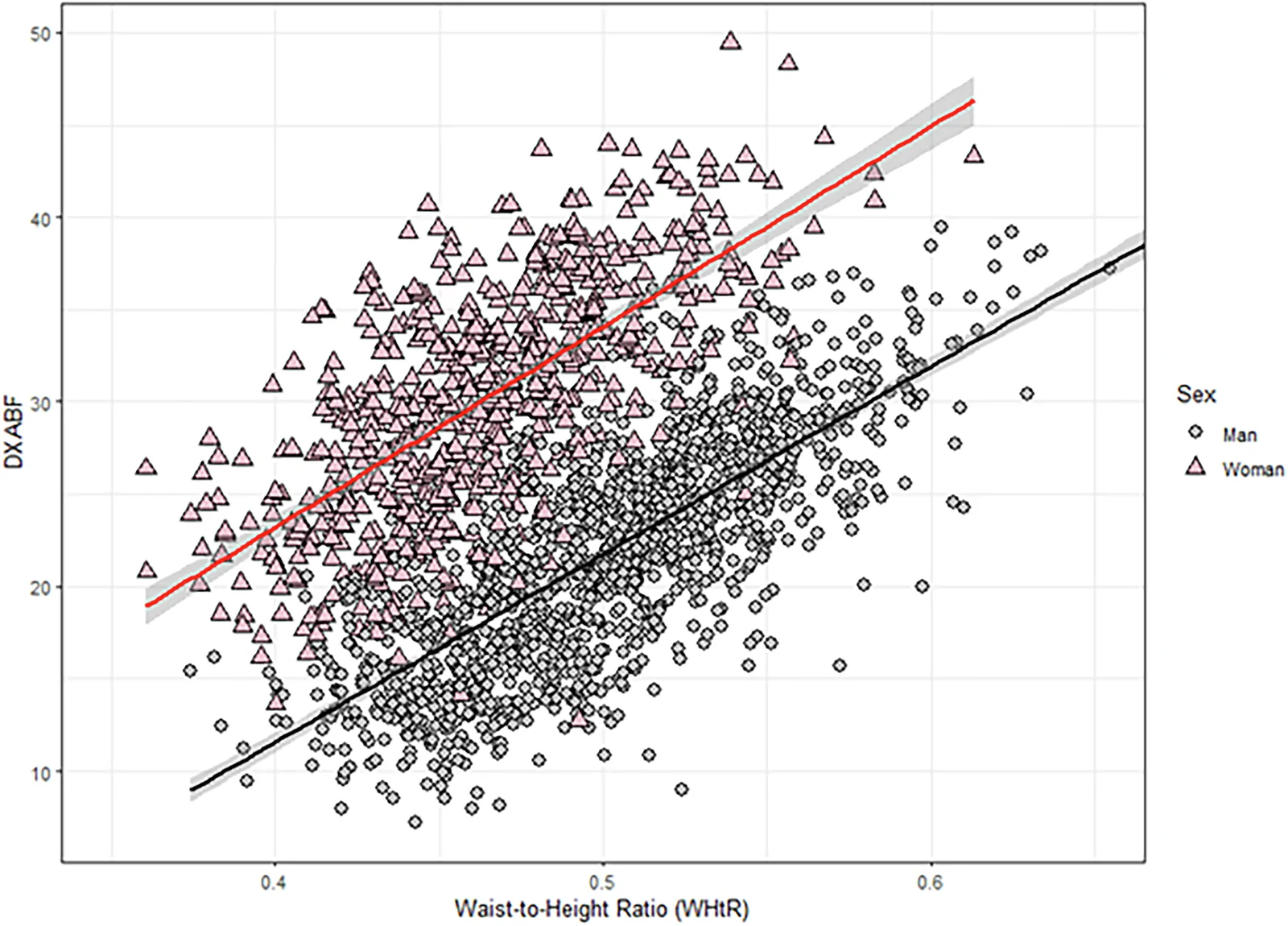
Relationship between Waist-to-Height Ratio (WHtR) and Body Fat Percentage. This scatter plot shows the positive correlation between WHtR and dual-energy X-ray absorptiometry (DXA) derived relative body fat percentage (DXA %BF). The relationship is shown for both men (black circles) and women (red triangles), with separate regression lines indicating the trend for each sex. For reference, at a WHtR of 0.50, the average DXA %BF is approximately 21% for men (black line) and 32% for women (red line); while at a WHtR of 0.55, the average DXA %BF increases to approximately 26% for men and 37% for women. This shows a consistent, positive linear relationship where %BF increases as the WHtR rises within this population of active adults.

**Fig. 3:**
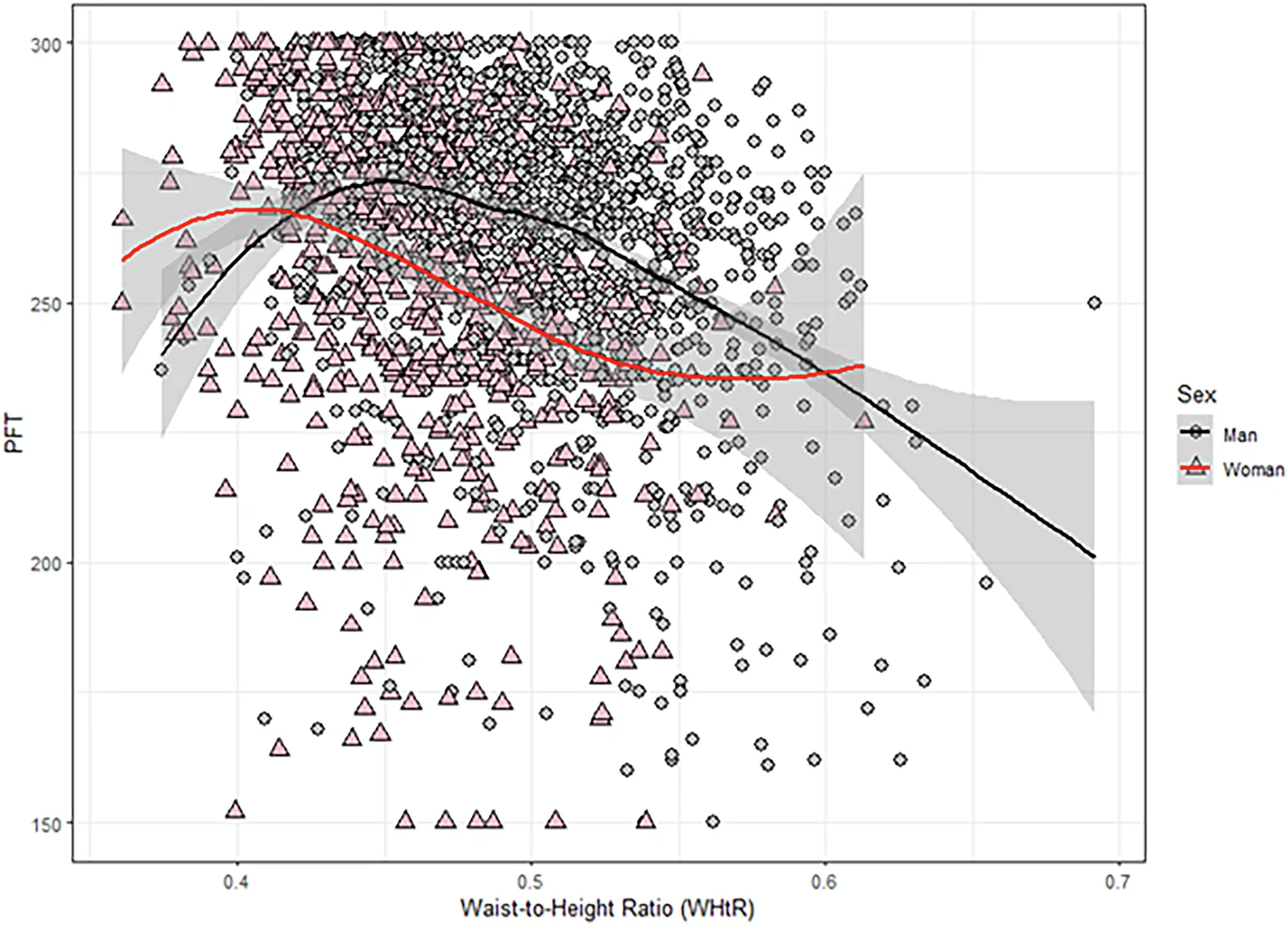
Relationship between Waist-to-Height Ratio (WHtR) and Physical Fitness Test (PFT) Scores. This scatter plot shows the relationship between WHtR and the PFT score. Data is shown for men (circles) and women (triangles), with a Locally Estimated Scatterplot Smoothing (LOESS) curve fitted for each sex to visualize the trend. The curves indicate that PFT scores remain relatively stable at lower WHtR values and then begin to decline as WHtR increases, particularly around a WHtR of 0.48–0.50.

**Fig. 4:**
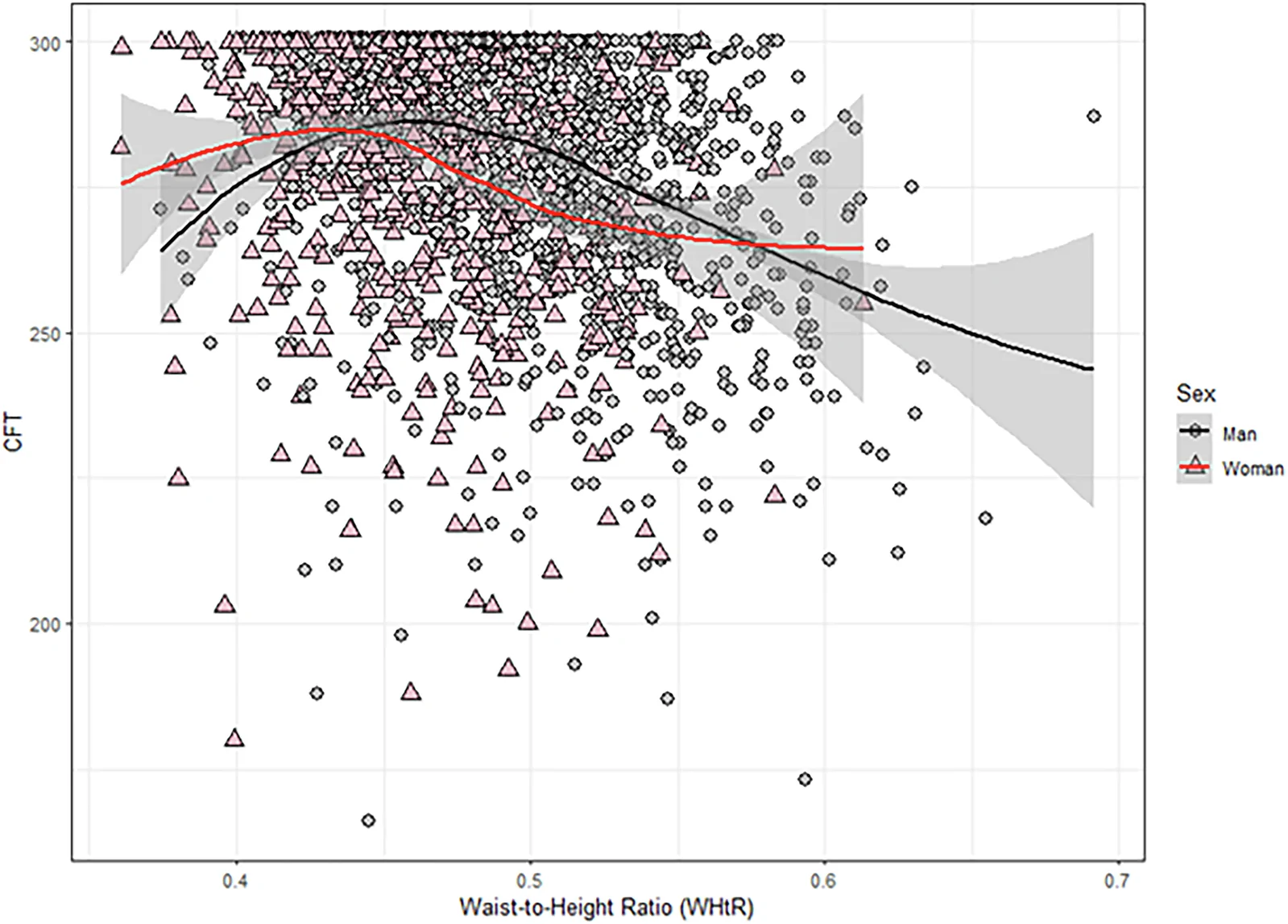
Relationship between Waist-to-Height Ratio (WHtR) and Combat Fitness Test (CFT) Scores. This scatter plot shows the relationship between WHtR and the CFT score. Data for men are shown as circles, and data for women are shown as triangles. A Locally Estimated Scatterplot Smoothing (LOESS) curve for each sex is included to illustrate the performance trend. Similar to the PFT scores, CFT scores show a notable decline as WHtR increases beyond the 0.48–0.50 range.

There was a strong positive correlation between WHtR and DXA %BF in both men (*r* = 0.754) and women (*r* = 0.677) (Fig. 2). WHtR was moderately negatively correlated with PFT (*r* = −0.330 for men, *r* = −0.297 for women) and CFT (*r* = −0.302 for men, *r* = −0.225 for women). LOESS plots demonstrated relatively stable PFT and CFT performance at lower WHtR values, followed by a progressive decline beginning at approximately WHtR 0.48–0.50, suggesting a functional inflection point beyond which central adiposity may adversely affect performance for both sexes (Figs. 3 and 4).

### Classification accuracy

**Fig. 5:**
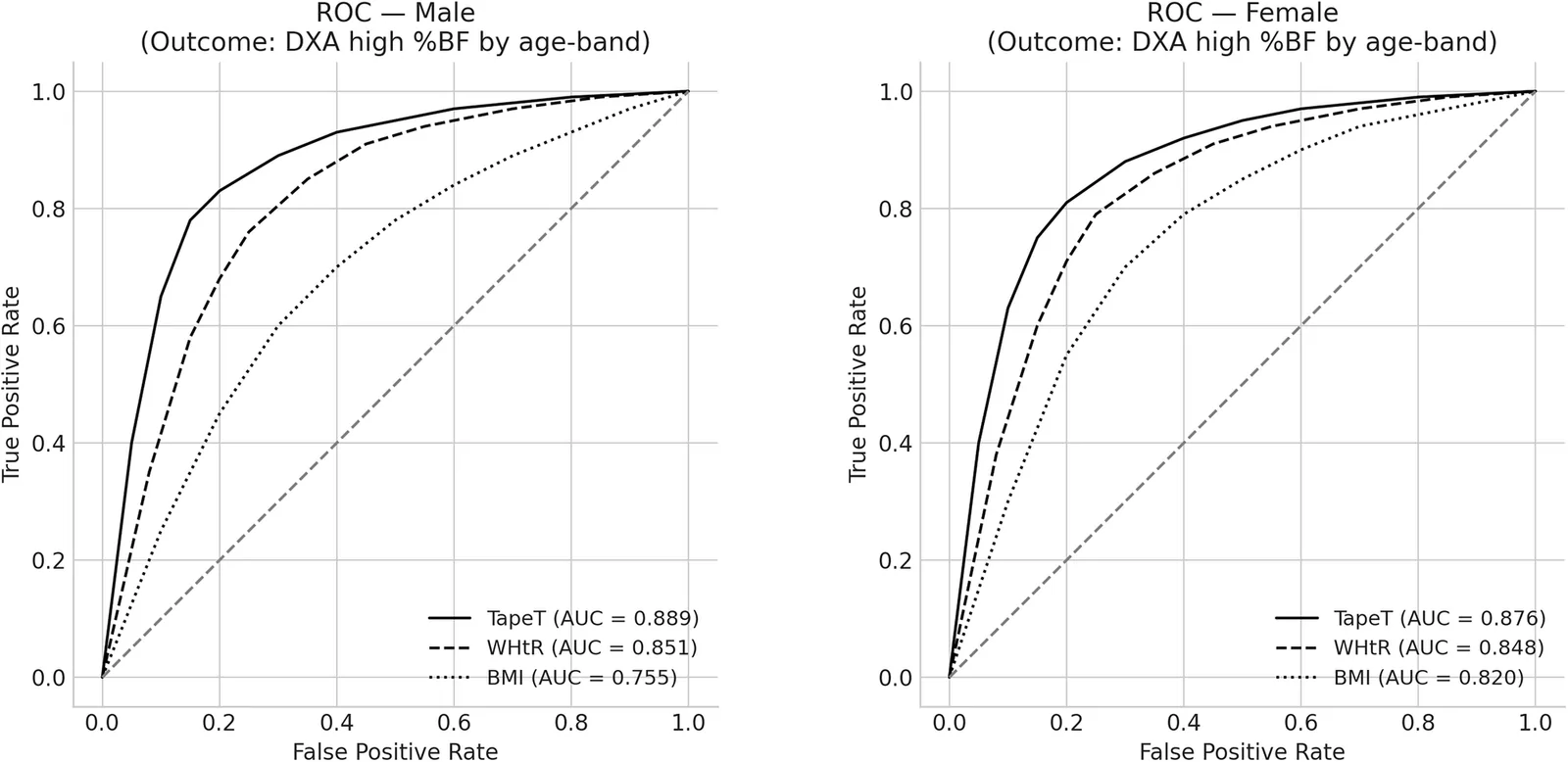
Performance of Anthropometric Measures in Predicting High Body Fat. These Receiver Operating Characteristic (ROC) curves compare the ability of Waist-to-Height Ratio (WHtR), Body Mass Index (BMI), and the circumference-based "Tape Test" (TT) to identify individuals with high percent body fat (%BF), as determined by DXA. The analysis is stratified by sex, with the left panel showing the results for men and the right panel for women. The Area Under the Curve (AUC) values are provided for each metric, indicating their respective predictive accuracy.

ROC analyses for predicting individuals exceeding age- and sex-specific %BF standards showed the TT had the highest AUC for both men (0.889) and women (0.876), followed by WHtR (0.851 and 0.848, respectively) and BMI (Fig. 5).

### Relationship to age

Multiple linear regression models controlling for age across all performance metrics (PFT, CFT) and body composition (DXA %BF) for both sexes showed that WHtR was an age-independent metric for both performance and body composition in this sample. When controlling for age, WHtR remained a highly significant negative predictor of both PFT and CFT scores for men and women (all *p* < 0.001). Age had a slight positive coefficient in these performance models, countering the possibility that the results were driven by younger individuals scoring higher. When modeling DXA %BF with both WHtR and Age as covariates, WHtR also remained the dominant predictor of DXA %BF (*p* < 0.001 for both sexes), while age accounted for virtually no variance in %BF over the contribution of WHtR (*p* = 0.044 for men; *p* = 0.481 for women) (Supplemental Table S1).

### Relationship between WHtR and individual fitness components

Analysis of individual fitness events revealed that WHtR had the strongest and most consistent relationship with aerobic and body weight-strength tasks. For both men and women, WHtR demonstrated a moderate positive correlation with run times for the 880-yard sprint (*r* = 0.415 and *r* = 0.299, respectively) and the 3-mile run (*r* = 0.375 and *r* = 0.290, respectively). These findings indicate that a higher WHtR is associated with slower (worse) performance in endurance running. Similarly, a weak-to-moderate negative correlation was found with pull-up repetitions for both men (*r* = −0.263) and women (*r* = −0.238), signifying that individuals with a higher WHtR performed fewer repetitions. In contrast, events focused on localized muscular endurance, such as crunches (*r* = −0.138 for men, *r* = −0.164 for women) and ammo can lifts (*r* = −0.069 for men, *r* = −0.001 for women), showed weak to negligible linear correlations with WHtR.

**Fig. 6:**
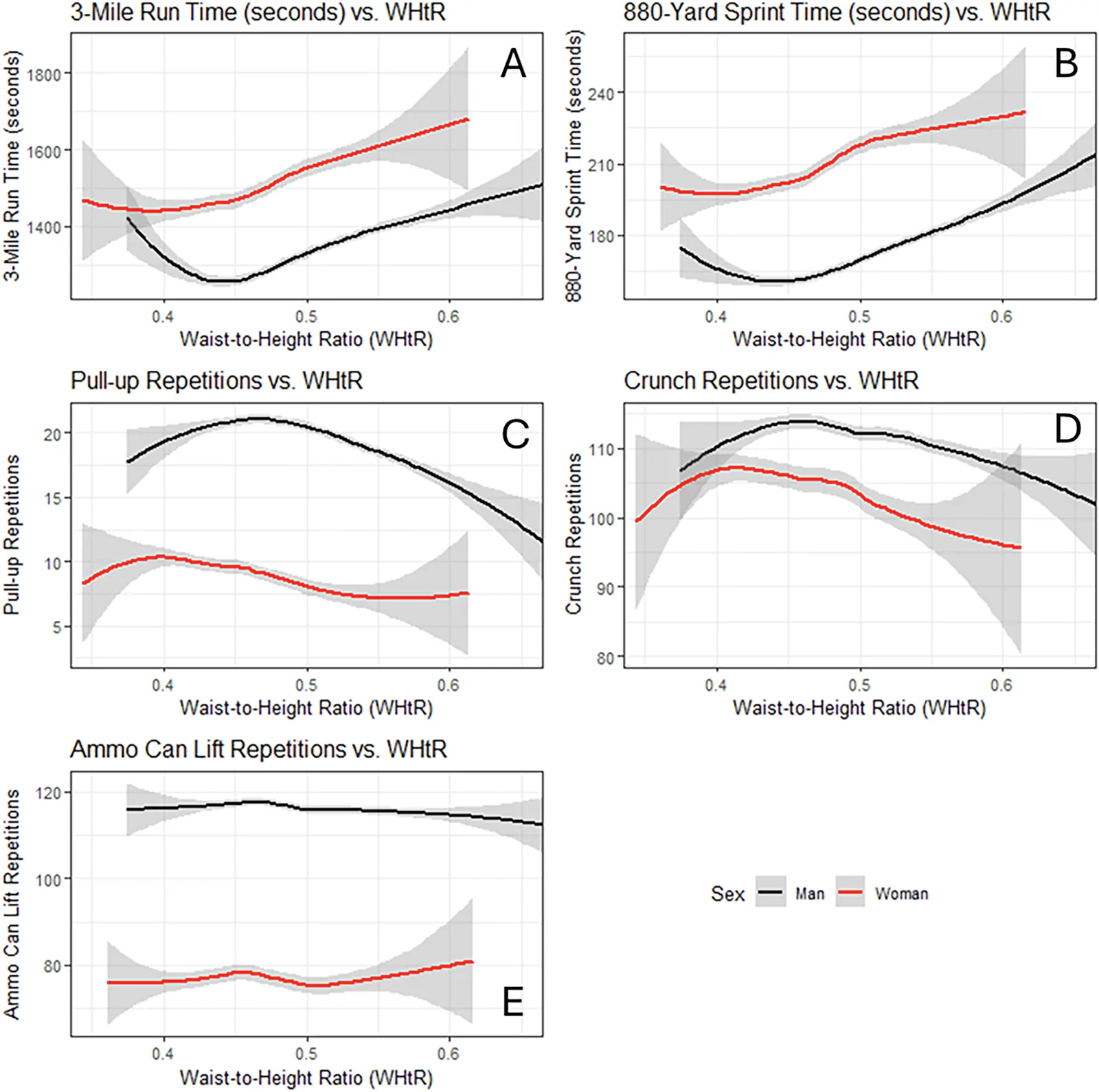
Relationship between Waist-to-Height Ratio (WHtR) and Individual Fitness Events. This figure shows the sex-specific relationship between WHtR and performance on five individual fitness events, visualized with Locally Estimated Scatterplot Smoothing (LOESS) curves. The panels display the following: **A** 3-Mile Run Time: Performance (in seconds) tends to worsen (increase) as WHtR increases. **B** 880-Yard Sprint Time: Sprint time (in seconds) shows a similar trend of increasing with higher WHtR. **C** Pull-up Repetitions: The number of repetitions decreases as WHtR increases. **D** Crunch Repetitions: Performance remains relatively flat across the WHtR spectrum. **E** Ammo Can Lift Repetitions: Performance shows little to no correlation with WHtR. In each panel, data are separated by sex, with black lines representing men and red lines representing women.

Visual analysis of the relationship between WHtR and individual performance confirmed these correlations and provided deeper insight into non-linear trends (Fig. 6). For the aerobically demanding events (the 3-mile run and the 880-yard sprint), performance (run time) showed a clear and progressive decline as WHtR increased. This decline appeared to accelerate for WHtR values above approximately 0.48–0.50, particularly in men (Fig. 6A, B). A similar pattern was observed for pull-up repetitions, where performance remained relatively stable at lower WHtR values before declining more steeply as WHtR surpassed 0.48–0.50 (Fig. 6C). In contrast, the LOESS curves for more localized muscular endurance events, such as crunches and ammo can lifts, were largely flat across the entire WHtR spectrum, indicating no clear performance trend (Fig. 6D, E). Furthermore, the weak linear correlation observed for events like crunches (*r* = −0.046 for men) is likely influenced by ceiling effects inherent to capped repetition scoring. The PFT has a maximum score for this event, and a large proportion of participants may have stopped upon reaching the maximum allowable repetitions, masking a potentially stronger underlying relationship between central adiposity and core muscular endurance.

### Subgroup and concordance analyses

Distribution-based comparisons showed that as WHtR increased, the proportion of individuals failing BMI and %BF standards and scoring in lower PFT/CFT classes increased (Supplementary Tables S2 and S3). An analysis of assessment method concordance showed that a WHtR threshold of 0.50 would identify a different subset of personnel for secondary screening compared to current BMI or TT limits, notably reducing the number of women requiring further assessment compared to the TT alone (Supplemental Table S4).

## Discussion

This study demonstrates that WHtR is a valuable indicator of physical performance, providing a clear, evidence-based rationale for its use as a readiness screener. The principal finding is the inverse relationship between WHtR and physical fitness scores, with a notable decline observed at a WHtR between 0.48 and 0.50. This observation aligns closely with the extensive evidence from civilian populations that establishes a WHtR of 0.50 as the threshold for early risk detection of cardiometabolic disease [15]. This convergence of performance and health data forms the basis for our primary recommendation to adopt a WHtR screening threshold of 0.50.

Although this threshold was more evident for men than for women, 0.5 is a practical screening threshold to identify individuals likely to be over the fat standards for age and sex and has been associated with increased disease risk in both men and women in many other studies [9–12]. The greater variation in body fat distribution in women likely complicates the statistical relationships between fitness, fatness, and WHtR in this study. Nevertheless, the individuals with greater risk of obesity tend to reflect this in male-type abdominal obesity that would be correctly assessed by WHtR [24]. This threshold serves as an effective, data-driven trigger to identify Marines who warrant a more comprehensive assessment of body composition due to potential health and performance risks associated with higher central adiposity.

The strong association with running and pull-up performance is physiologically consistent, as these activities require the individual to move their own body mass against gravity or over a distance. Excess mass, including central adiposity captured by WHtR, reflects a direct physical impediment to performance in these critical tasks. Conversely, the lack of a strong correlation with more isolated exercises like crunches is equally informative. It suggests that WHtR is less indicative of localized core muscle endurance and more representative of systemic factors affecting gross motor performance. This distinction is important, as it implies that while WHtR is a powerful screening tool for overall physical readiness and load-bearing activities, it should not be misinterpreted as a direct measure of an individual’s core strength or specific muscular capabilities. These findings further support using WHtR as a useful screener rather than as a diagnostic tool for specific physical capabilities.

Furthermore, our performance-based findings align remarkably well with the established health-risk framework for WHtR, which is best understood as a continuum of risk. While our data identified a performance inflection point near 0.50, civilian population research designates a 0.50 WHtR as the primary ‘prevention threshold’ for cardiometabolic disease. The risk escalates as WHtR increases; values in the 0.52–0.53 range are considered a ‘diagnostic threshold,’ indicating a high probability of established metabolic syndrome, insulin resistance, or dyslipidemia [25]. Higher thresholds, such as 0.55 or above, demonstrate even greater specificity for identifying high-risk individuals [26]. In this study, we identified a decline in physical performance occurring at 0.48 to 0.50 WHtR. In recent analyses of the same military population, the mean WHtR for “top-tier” physically fit Marines that recorded maximum (perfect) scores for their fitness evaluations was 0.45 [27], while a very selective group of young officers aged 21-30 had a mean WHtR of 0.46, both values well below the 0.50 cutoff [28]. This finding establishes a healthy baseline for this specific cohort and reinforces that a WHtR of 0.50 represents a meaningful deviation from the norm, providing further support for its use as an effective initial screening value to detect emerging risk.

The non-linear trends visualized in the LOESS plots (Figs. 3, 4, and 6) strongly suggest the existence of a practical performance threshold. The clear inflection point observed around a WHtR of 0.50 for running and pull-up performance aligns with the threshold where central adiposity may begin to have a mechanically and metabolically significant impact on load-bearing and aerobic efficiency. The visual evidence from these plots provides a compelling rationale for the 0.50 screening cut-point, as it appears to be the point at which performance in operationally relevant tasks begins to meaningfully degrade.

While WHtR is a practical screening tool, it is not a direct proxy for %BF. Our results confirm that the TT is a more accurate predictor of DXA-measured %BF. Therefore, a two-step process is recommended: a simple WHtR screen followed by a more accurate secondary assessment for those who exceed the threshold. This approach aligns with modern best practices, ensuring that muscular, high-performing Marines are not incorrectly penalized by a screening-level metric while efficiently targeting resources toward at-risk individuals. It would be redundant to follow a waist circumference screening metric with a waist circumference-based TT assessment standard. Multi-frequency bioelectrical impedance analysis (MF-BIA) represents a practical example of a secondary assessment option, recognizing that device availability, standardization, and operator training are important considerations for large-scale implementation [29–31]. Recent evaluations have confirmed that modern MF-BIA systems demonstrate high methodological reliability and consistent day-to-day measurements for body composition components under controlled conditions, making them suitable for this role [30].

While our analysis is specific to a cohort of U.S. Marines, the physiological principles are broadly applicable, and the regulations already permit implementation of WHtR [32]. The observed inverse relationship between central adiposity (via WHtR) and performance in load-bearing tasks like running and pull-ups is not unique to the military. Other physically demanding fields, i.e., firefighting and police tactical units, would likely see similar performance decrements with increasing WHtR. The proposed 0.50 WHtR threshold could therefore serve as a practical, evidence-based initial screening tool for these occupations, identifying individuals who may warrant further fitness and health assessment. Furthermore, these findings can inform fitness standards in some collegiate and professional sports, where body composition is closely linked to athletic performance.

This study is limited by its cross-sectional design, which precludes causal inferences between WHtR and performance changes over time. Future longitudinal research should track changes in WHtR, body composition, and fitness to establish stronger causal links. Further investigation is also needed to determine optimal WHtR cut-points for identifying cardiometabolic disease risk in this population and to conduct a cost-benefit analysis of large-scale MF-BIA implementation.

## Conclusions

Screening tests serve as a tool to identify individuals who may require further assessment for obesity-related health and performance outcomes. In this context, this study provides an evidence-based recommendation for implementing WHtR as the Marine Corps’ primary body composition screening tool. Replacement of body weight with a waist circumference metric is a positive step towards more specific and accurate physical readiness standards. The relationship between higher WHtR and lower physical fitness scores supports adopting a WHtR of 0.50 as the official screening standard. Marines exceeding this threshold may benefit from a secondary assessment using MF-BIA to determine their %BF. This two-step process effectively identifies personnel at risk for poor health and performance outcomes while ensuring that muscular, high-performing Marines are assessed fairly, thereby establishing a data-driven standard for maintaining force readiness and health.

## Supplementary information

Supplemental Materials

## Data Availability

Data and analyses from the current study are available from the corresponding author on reasonable request.
