# Supplementary material for: A waist-to-height ratio of 0.50 marks declining physical performance in active adults
Source: Eur J Clin Nutr. 2026 Jul 30;80(10):1096–104. doi: 10.1038/s41430-026-01798-z (PMC13690569; doi:10.1038/s41430-026-01798-z)
Supplement: Supplementary file 1 [file 41430_2026_1798_MOESM1_ESM.docx]

**Supplemental Materials**

**Table S1.** Age-Adjusted Multiple Linear Regression Models Predicting Fitness Scores and Body Fat (n = 2,153; 1,421 men; 732 women).

**Table S2.** Men distribution-based comparisons for relationships of body mass index (BMI), dual-energy X-ray absorptiometry relative body fat (DXA %BF), and physical and combat fitness test (PFT, CFT) scores to grouped ranges (bins) of waist-to-height ratio (WHtR) (n = 1,339).

**Table S3.** Women distribution-based comparisons for relationships of body mass index (BMI), dual-energy X-ray absorptiometry relative body fat (DXA %BF), and physical and combat fitness test (PFT, CFT) scores to grouped ranges (bins) of waist-to-height ratio (WHtR) (n = 683).

**Table S4.** Comparison of Personnel Exceeding Body Composition and Anthropometric Thresholds by Sex and Age Group (n = 2,022; 1,339 men; 683 women).

**Table S1.** Age-Adjusted Multiple Linear Regression Models Predicting Fitness Scores and Body Fat (n = 2,153; 1,421 men; 732 women).

| Dependent Variable | Sex | WHtR Coefficient, β | WHtR  *p*-value | Age Coefficient, β | Age  *p*-value | Model Adjusted R^2^ |
| --- | --- | --- | --- | --- | --- | --- |
| PFT | **Men** | -238.33 | < 0.001 | 0.88 | < 0.001 | 0.166 |
|  | **Women** | -237.20 | < 0.001 | 0.54 | < 0.001 | 0.100 |
| CFT | **Men** | -161.93 | < 0.001 | 0.44 | < 0.001 | 0.114 |
|  | **Women** | -125.50 | < 0.001 | 0.19 | 0.088 | 0.051 |
| DXABF (%BF) | **Men** | 103.16 | < 0.001 | -0.03 | 0.044 | 0.568 |
|  | **Women** | 108.66 | < 0.001 | -0.01 | 0.481 | 0.457 |

**Table S2.** Men distribution-based comparisons for relationships of body mass index (BMI), dual-energy X-ray absorptiometry relative body fat (DXA %BF), and physical and combat fitness test (PFT, CFT) scores to grouped ranges (bins) of waist-to-height ratio (WHtR) (n = 1,339).

| **Variable** | **Value** | **WHtR (binned by 0.02 increments)** | | | | | | | | | | | | | | | | |
| --- | --- | --- | --- | --- | --- | --- | --- | --- | --- | --- | --- | --- | --- | --- | --- | --- | --- | --- |
|  |  | 0.36, 0.38 | 0.38, 0.40 | 0.40, 0.42 | 0.42, 0.44 | 0.44, 0.46 | 0.46, 0.48 | 0.48, 0.50 | 0.50, 0.52 | 0.52, 0.54 | 0.54, 0.56 | 0.56, 0.58 | 0.58, 0.60 | 0.60, 0.62 | 0.62, 0.64 | 0.64, 0.66 | 0.66, 0.68 | 0.68, 0.70 |
| **BMI** | ≤27.5 | 1 | 5 | 26 | 84 | 140 | 164 | 137 | 87 | 39 | 13 | 3 | - | - | - | - | - | - |
|  | >27.5 | - | - | - | - | 5 | 26 | 78 | 129 | 148 | 129 | 64 | 41 | 13 | 5 | 1 | - | 1 |
| **DXA %BF** | ≤18 | 1 | 5 | 23 | 62 | 112 | 90 | 66 | 28 | 7 | 4 | 1 | - | - | - | - | - | - |
|  | >18 | - | - | 3 | 22 | 33 | 100 | 149 | 188 | 180 | 138 | 66 | 41 | 13 | 5 | 1 | - | 1 |
| **PFT** | 1st –235-300 | 1 | 5 | 22 | 79 | 136 | 176 | 201 | 197 | 167 | 111 | 48 | 31 | 6 | - | - | - | 1 |
|  | 2nd - 200-234 | - | - | 2 | 4 | 7 | 11 | 13 | 17 | 15 | 22 | 12 | 6 | 4 | 2 | - | - | - |
|  | 3rd - 150-199 | - | - | 2 | 1 | 2 | 3 | 1 | 2 | 5 | 9 | 7 | 4 | 3 | 3 | 1 | - | - |
| **CFT** | 1st –235-300 | 1 | 5 | 26 | 79 | 142 | 188 | 210 | 211 | 181 | 132 | 63 | 37 | 10 | 3 | - | - | 1 |
|  | 2nd - 200-234 | - | - | - | 4 | 1 | 2 | 5 | 4 | 6 | 9 | 4 | 3 | 3 | 2 | 1 | - | - |
|  | 3rd - 150-199 | - | - | - | 1 | 2 | - | - | 1 | - | 1 | - | 1 | - | - | - | - | - |

**Table S3.** Women distribution-based comparisons for relationships of body mass index (BMI), dual-energy X-ray absorptiometry relative body fat (DXA %BF), and physical and combat fitness test (PFT, CFT) scores to grouped ranges (bins) of waist-to-height ratio (WHtR) (n = 683).

| **Variable** | **Value** | **WHtR (binned by 0.02 increments)** | | | | | | | | | | | | | | | | |
| --- | --- | --- | --- | --- | --- | --- | --- | --- | --- | --- | --- | --- | --- | --- | --- | --- | --- | --- |
|  |  | 0.36, 0.38 | 0.38, 0.40 | 0.40, 0.42 | 0.42, 0.44 | 0.44, 0.46 | 0.46, 0.48 | 0.48, 0.50 | 0.50, 0.52 | 0.52, 0.54 | 0.54, 0.56 | 0.56, 0.58 | 0.58, 0.60 | 0.60, 0.62 | 0.62, 0.64 | 0.64, 0.66 | 0.66, 0.68 | 0.68, 0.70 |
| **BMI** | ≤26 | 6 | 20 | 64 | 98 | 112 | 61 | 23 | 6 | 2 | - | - | - | - | - | - | - | - |
|  | >26 | 1 | - | 2 | 9 | 22 | 68 | 79 | 46 | 42 | 17 | 2 | 2 | 1 | - | - | - | - |
| **DXA %BF** | ≤27 | 7 | 18 | 47 | 56 | 54 | 31 | 8 | 1 | - | 1 | - | - | - | - | - | - | - |
|  | >27 | - | 2 | 19 | 51 | 80 | 98 | 94 | 51 | 44 | 16 | 2 | 2 | 1 | - | - | - | - |
| **PFT** | 1st –235-300 | 7 | 17 | 61 | 92 | 110 | 107 | 76 | 39 | 25 | 12 | 1 | 1 | - | - | - | - | - |
|  | 2nd - 200-234 | - | 2 | 3 | 12 | 16 | 19 | 19 | 12 | 10 | 4 | 1 | 1 | 1 | - | - | - | - |
|  | 3rd - 150-199 | - | 1 | 2 | 3 | 8 | 3 | 7 | 1 | 9 | 1 | - | - | - | - | - | - | - |
| **CFT** | 1st –235-300 | 7 | 17 | 65 | 103 | 131 | 125 | 95 | 51 | 39 | 15 | 2 | 1 | 1 | - | - | - | - |
|  | 2nd - 200-234 | - | 2 | 1 | 4 | 2 | 4 | 6 | 1 | 4 | 2 | - | 1 | - | - | - | - | - |
|  | 3rd - 150-199 | - | 1 | - | - | 1 | - | 1 | - | 1 | - | - | - | - | - | - | - | - |

**Table S4.** Comparison of Personnel Exceeding Body Composition and Anthropometric Thresholds by Sex and Age Group (n = 2,022; 1,339 men; 683 women).

| **Sex** | **Metric** | **17-26 years** | **27-39 years** | **40-45 years** | **46+ years** |
| --- | --- | --- | --- | --- | --- |
| **Men** | **Sample size** | 511 | 646 | 125 | 57 |
|  | **Exceed Tape Limit (>18%, 19%, 20%, 21%)** | 190 | 357 | 84 | 30 |
|  | **Exceed BMI Limit (>27.5)** | 196 | 343 | 73 | 28 |
|  | **Exceed Both Tape & BMI** | 155 | 282 | 64 | 28 |
|  | **Exceed Both Tape & WHtR (>0.50)** | 170 | 351 | 88 | 35 |
| **Women** | **Sample size** | 293 | 322 | 58 | 10 |
|  | **Exceed Tape Limit (>27%, 28%, 29%, 30%)** | 210 | 230 | 35 | 7 |
|  | **Exceed BMI Limit (>26)** | 121 | 146 | 21 | 3 |
|  | **Exceed Both Tape & BMI** | 121 | 145 | 21 | 3 |
|  | **Exceed Both Tape & WHtR (>0.50)** | 53 | 54 | 10 | 1 |
